# Metabolomic Strategy for Studying the Intervention and the Synergistic Effects of the Shexiang Baoxin Pill for Treating Myocardial Infarction in Rats

**DOI:** 10.1155/2013/823121

**Published:** 2013-02-28

**Authors:** Li Xiang, Peng Jiang, Shuping Wang, Yaohua Hu, Xiaojun Liu, Rongcai Yue, Weidong Zhang, Runhui Liu

**Affiliations:** ^1^School of Pharmacy, Second Military Medical University, No. 325 Guohe Road, Shanghai 200433, China; ^2^School of Pharmacy, Shanghai Jiao Tong University, Shanghai 200030, China; ^3^Department of Pharmacognosy, King Saud University, Riyadh 11451, Saudi Arabia

## Abstract

A metabolomic approach has been developed for evaluating the therapeutic effects of the bioactive components and the synergistic efficacy of the Shexiang Baoxin Pill (SBP) on myocardial infarction (MI) in rats. The MI rats were administered the SBP, muscone, cinnamic acid, bufalin, ginsenoside Re, ginsenoside Rb1, cholic acid, borneol, and a combined version of these bioactive components (SFSBP). Liquid chromatography/quadrupole time-of-flight mass spectrometry (LC-Q-TOF/MS) was used to obtain the mass data from the rats' serum. The number of biomarkers that were reversed by SFSBP was greater than any of the monotherapy groups. The PLS-DA score plots demonstrated that the SFSBP group results were located closer to the sham group than any of the monotherapy groups and that the SBP group was located closer to the sham group than the SFSBP treatment group. The reversing results observed with SFSBP showed synergistic effects when compared with those of the individual bioactive components that were used as monotherapy. Meanwhile, the SBP displayed superior regulation efficacy to SFSBP in MI rats, indicating that there must be other active components in the SBP that were responsible for the treatment of MI that were not included in the SFSBP treatment.

## 1. Introduction

Traditional Chinese Medicines (TCMs) have a long-standing use in clinical practice, and their effectiveness has also been confirmed [1–5]. TCMs hold an important position in the health care tradition of many countries, particularly in Asia [6]. As each TCM employs a large number of chemical compounds and as these components are its pharmacodynamic material basis, TCM itself is definitely a multicomponent agent. Characteristically, TCMs focus on body's response to pathogenic factors more than on the pathological mechanisms, and TCM regulates them in a more holistic way with minimal side effects [7]. Although the therapeutic effectiveness of TCM has been recognized significantly for thousands of years, its synergistic effects have not been sufficiently clarified. 

The Shexiang Baoxin Pill (SBP), which consists of seven herbal medicines, including *Moschus*, *Radix Ginseng*, *Calculus Bovis, Styrax*, *Cortex Cinnamomi*, *Venenum Bufonis*, and* Borneolum Syntheticum*, has been widely used for treating coronary heart disease (CHD) in China for many years [8–12]. The therapeutic mechanisms of the SBP have been studied in our lab for approximately ten years. Although Peng Jiang et al. [13] and Li Xiang et al. [14] have demonstrated that the therapeutic mechanisms of the SBP were involved mainly in inhibiting dysfunctions in energy metabolism, oxidative injury, and inflammation in the development of myocardial infarction (MI), the advantages of the synergistic efficacy of the SBP had not been investigated. There are hundreds of compounds in the SBP, but most of them are nonbioactive ingredients. In other words, they are not useful therapeutically. Therefore, we hope to rebuild a simplified formula of an SBP (SFSBP) using the bioactive ingredients to attempt to clarify the advantages of the synergistic efficacy of the SBP.

It has been reported that there are hundreds of compounds in the SBP [15, 16]. Compounds such as muscone, cinnamic acid, bufalin, ginsenoside Re, ginsenoside Rb1, cholic acid, and borneol are the principal active components in the ingredients (*Moschus*, *Cortex Cinnamomi*, *Styrax*, *Venenum Bufonis*, *Radix Ginseng*, *Calculus Bovis*, and *Borneolum Syntheticum*), and they are also responsible for the therapeutic effectiveness of the SBP [17]. In this study, these 7 bioactive compounds were combined into a simplified formula of the Shexiang Baoxin Pill (SFSBP) according to their proportions in the SBP [15, 16]. 

Metabolomics is a systematic approach that can be used for understanding processes ranging from the analysis of metabolic profiles *in vivo* to the dynamic responses to pathophysiological stimuli or drug interventions [18]. It provides insights into the global metabolic condition of the entire organisms, which is reasonably coincident with the systemic and integrative nature of TCM [19]. Metabolomics has been applied to the study of the potential mechanisms of many TCMs, such as the Shuanglong formula [20], the Sini Decoction [21], and the Compound Danshen Tablets [22, 23]. 

As part of an ongoing study, a metabolomic methodology has been employed to investigate the synergistic efficacy of the multiple components of SBP in MI rats. Through a comparative study of the holistic intervention effects of the SBP, the SFSBP, and the 7 single bioactive compounds on MI rats, the synergistic efficacy of the multicomponent properties of the SBP has been clarified preliminarily. 

## 2. Experiment 

### 2.1. Materials and Reagents

HPLC grade methanol and acetonitrile were purchased from Honeywell (NJ, USA). Spectroscopic grade formic acid and leucine enkephalin were purchased from Sigma-Aldrich (St. Louis, MO, USA). Ultrapure water was prepared with a Milli-Q water purification system (MA, USA). The assay kits used for creatine kinases (CK) and lactate dehydrogenase (LDH) were purchased from Nanjing Jiancheng Bioengineering Institute (Nanjing, China). The Shexiang Baoxin Pill (SBP) was kindly donated by Shanghai Hutchison Pharmaceuticals (Shanghai, China). Muscone, cinnamic acid, bufalin, ginsenoside Re, ginsenoside Rb1, cholic acid, and borneol (purity > 98%) were purchased from Shanghai Ronghe Biopharmaceutical Co., LTD (Shanghai, China). 

### 2.2. Animals

Eighty male Sprague-Dawley rats (200 ± 20 g) were purchased from SLAC Laboratory Animal Co., Ltd (Shanghai, China). All of the animals were kept in an animal room with a constant temperature of 23 ± 2°C and a 12 h dark-light cycle with free access to food and water. The animal facilities and protocols were used with the permission of the Institutional Animal Care and Use Committee of the Second Military Medical University. All of the animals were treated with humane care throughout the experiment under the previous conditions for a 2-week acclimation period. 

### 2.3. MI Model and Drug Administration

An MI model was induced by the left anterior descending coronary artery (LADCA) ligation [24, 25]. First, animals were anesthetized with ether, immobilized on a pad and positioned on their backs. Then, the heart was exteriorized immediately after thoracotomy in the fourth left intercostal space. After the heart was returned to its normal position, a 4–0 black silk ligature was securely ligated in the MI group rats. To ascertain that the MI model had been successful, electrocardiograms (ECGs) were recorded using a MPA 2000 biosignal analysis system (Alcott Biotech Co. Ltd., Shanghai, China) to obtain an abnormal Q wave (less than 0.3 mV). The sham group was operated upon using the same process as previously mentioned, except for the ligation step. In total, 64 MI rats and 8 sham rats survived. 

Fifty-six MI rats were randomly grouped and treated with 9 types of medicines: SBP (28 mg/kg/day, *n* = 7), SFSBP (40 mg/kg/day, *n* = 7, composed of muscone (2.28 mg), cinnamic acid (2.57 mg), bufalin (1.16 mg), ginsenoside Re (7.87 mg), ginsenoside Rb1 (3.20 mg), cholic acid (10.20 mg), and borneol (12.72 mg)), muscone (150 mg/kg/day, *n* = 6), cinnamic acid (50 mg/kg/day, *n* = 6), bufalin (10 mg/kg/day, *n* = 6), ginsenoside Re (20 mg/kg/day, *n* = 6), ginsenoside Rb1 (40 mg/kg/day, *n* = 6), cholic acid (80 mg/kg/day, *n* = 6), or borneol (100 mg/kg/day, *n* = 6). The nine types of medicines were ground into fine powders and dissolved in a 0.5% carboxymethyl cellulose sodium salt (CMC-Na) aqueous solution. The remaining 8 MI rats were treated as an MI model group, and 0.5% CMC-Na was orally administered, as was performed for the 8 sham rats. All of the rats were orally gavaged for 15 consecutive days.

### 2.4. Sample Collection

Blood was collected from the ophthalmic venous plexus after all the rats were anaesthetized with ether on the 15th day. The blood was then centrifuged at 3,500 rpm for 10 min at 4°C, 1 h after the blood collection. Then, 200 *μ*L of the supernatant serum obtained was collected and stored at −20°C for analysis of CK and LDH. The remaining serum (>200 *μ*L) was stored in a −80°C refrigerator for LC-Q-TOF/MS analysis. The hearts of 72 rats were excised and immediately fixed in 10% formalin for the analysis of the histopathology.

### 2.5. Preparation of Metabolomic Samples

The serum samples were thawed before analysis. Each 100 *μ*L of serum aliquot was added to 1000 *μ*L of methanol. The mixture was vortexed for 2 min and centrifuged at 12,000 rpm for 10 min at 4°C. The supernatant was stored at −80°C for at least 24 hours before analysis. 

The blank sample was acetonitrile, and it was run after every two serum sample injections to minimize carryover. Equal volumes of methanol and ultrapure water were mixed as a needle cleaning phase and were washed for 1 min during every injection.

A volume of 10 *μ*L was pooled as a quality control (QC) sample from each serum sample. The preparation process for the QC samples was the same as for the analysis samples. The QC samples were injected randomly during the analysis sequence to ensure the stability of the instrument that was used to evaluate the mass, the retention time, and the ionic intensity.

### 2.6. LC-Q-TOF/MS Analysis

Metabolomic analysis was performed on an Agilent-1200 LC system coupled to an Agilent-6520 Q-TOF mass spectrometer (Agilent Technologies, Palo Alto, CA, USA) and equipped with an electrospray ionization source. Separation of all of the serum samples was performed on an Eclipse plus C_18_ column (1.8 *μ*m, 2.1 mm × 100 mm, Agilent). The column temperature was maintained at 45°C. Ultrapure water (a) with 0.1% formic acid and acetonitrile (b) with 0.1% formic acid were used as the mobile phase, and the flow rate was set at 0.25 mL/min. The gradient program was as follows: 0–1.5 min, 2% B; 1.5–9 min, 100% B; 9–11 min, 85% B; 11–13 min, 80% B; 13–18 min, 2% B; 18–22 min, 2% B in positive mode and 0–1.5 min, 2% B; 1.5–9 min, 85% B; 9–12 min, 100% B; 12–15 min, 100% B; 15–19 min, 2% B; 19–23 min, 2% B in negative mode. The sample injection volume was 1 *μ*L.

The parameters for mass detection were set as follows: the gas temperature was 330°C; the drying gas (N_2_) flow rate was 8 L min^−1^; the nebulizer gas pressure was 35 psig, the Vcap was 3,700 V in the negative mode and 3,900 V in the positive mode; the skimmer was 65 V; the fragmentor was 160 V; and the mass scan range was *m/z* 50–1000. Leucine enkephalin was used as the lock mass (*m/z* 554.2615 in the negative mode and 556.2771 in the positive mode). The MS/MS analysis was acquired in the targeted MS/MS mode with three collision energies: 10 ev, 20 ev and 40 ev.

### 2.7. Method Validation

Validation of the method was carried out to confirm the suitability of long batch analysis. In this study, six extracted ions, including *m/z* 269.05 (homocysteine, positive mode), *m/z* 118.07 (L-valine, positive mode), *m/z* 282.27 (oleamide, positive mode), *m/z* 608.3631 (PGPC, negative mode), *m/z* 188.0768 (3-indolepropionic acid, negative mode), and *m/z* 165.046 (3-methylxanthine, negative mode), were chosen to assess the repeatability and stability of the established method by assessing the variations in peak areas and retention times.

Six parallel samples that were obtained from the same sample were prepared using the same extraction method and were injected continuously for the assessment of the repeatability. QC samples were injected after every 6 serum sample injections to evaluate the stability of the sequence analysis.

### 2.8. Data Processing

The LC-Q-TOF/MS data were analyzed by Agilent Mass Hunter Qualitative Analysis Software (Agilent Technologies, Palo Alto, CA, USA). The parameters for the data collection were set as follows: mass ranging from 50 to 1000 amu and retention time ranging from 0 to 12 min, and mass tolerance was 0.05 Da, retention time tolerance was 0.1 min, and peak relative height was ≥1.5%. Mass data (*m/z*) and retention time (*t*_*R*_) were listed as the identifiers for each peak. Before the multivariate analysis, the ion intensities for each peak were normalized to the total area to correct for the MS response shift through sequence analysis due to the long duration of this metabolomic study. Partial least squares discriminant analysis (PLS-DA) was performed through SIMCA-P (version 11.0, Umetrics, Umea, Sweden) for multivariate analysis. One-way analyses of variance (ANOVAs) were performed using a Bonferroni correction of the SPSS 13.0 package for Windows (SPSS Inc., Chicago, IL, USA) for the analysis of significance. Differences were considered significant at values of *P* < 0.05.

## 3. Results and Discussion

### 3.1. Pharmacology Study

#### 3.1.1. Serum Enzyme Measurements

**Figure 1:**
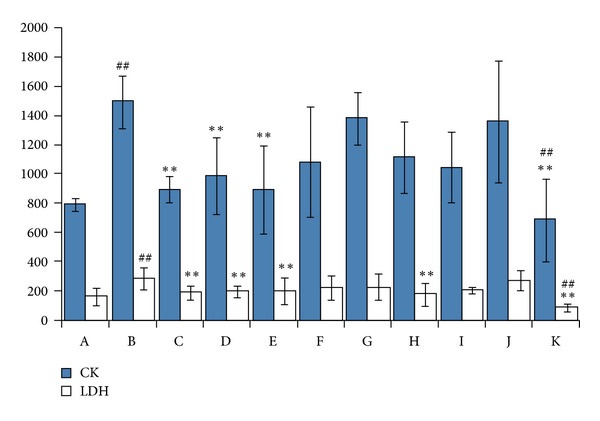
CK and LDH results in rat serum from MI group. LDH: lactate dehydrogenase and CK: and creatine kinases. (A) sham group, (B) MI group, (C) SBP group, (D) SFSBP group, (E) ginsenoside Re, (F) ginsenoside Rb1, (G) bufalin group, (H) cinnamic acid group, (I) muscone group, (J) borneol group, and (K) cholic acid group. **P < 0.01 versus MI group and ^##^ *P *< 0.01 versus sham group.

The activities of the serum enzymes CK and LDH are considered to be important parameters in the assessment of myocardial injury [26, 27]. In the present study, the serum concentrations of CK and LDH were measured to evaluate both the validity of the MI model and the therapeutic effectiveness of different treatments. As shown in Figure 1, the concentrations of CK and LDH were significantly increased (*P *< 0.01, compared with the sham group) in the MI group (B), which indicated that the MI model had been successfully induced. The cinnamic acid (H) group only acquired a significant restoration effect on LDH when compared with the MI group (*P *< 0.01). The cholic acid (K) group not only showed a significant difference from the MI group but also showed a significant difference when compared with the sham group (*P *< 0.01), suggesting that cholic acid had over-regulated the disturbed balance of LDH and CK *in vivo* that was induced by the MI and that there might be side effects during regulation. However, the reducing effects of the SBP (C), the SFSBP (D), and the ginsenoside Re (E) groups seemed more robust than did the remaining groups (*P *< 0.01, compared with the sham group). 

#### 3.1.2. Effects of MI on Myocardial Tissue

**Figure 2:**
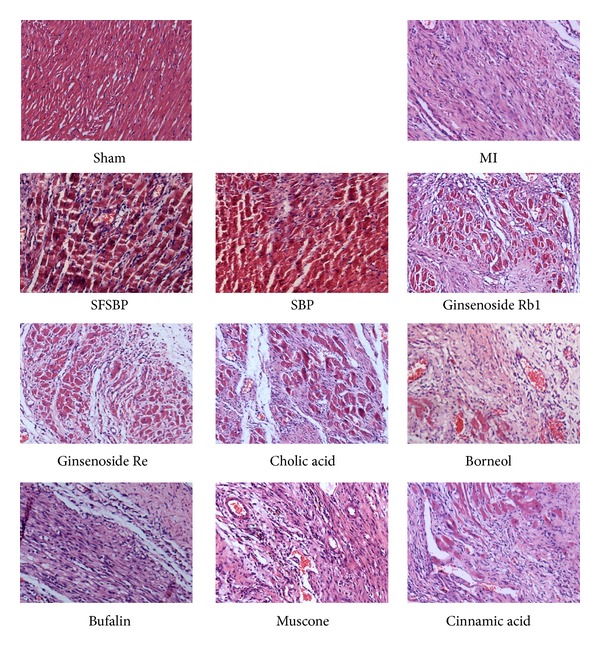
Myocardial tissues of 11 groups under light microscopy.

As shown in Figure 2, histological sections of myocardial tissue from the MI group can be differentiated clearly from those of the sham group. There was serious swelling of the fibroblasts and the fibroblastic hyperplasia in the MI group, while subendocardial necrosis, infiltrated leukocytes, chronic inflammatory cells, edema, and vacuoles could be clearly observed. However, these symptoms, induced by myocardial injury, were almost completely lacking in the sham group. Histopathological examination of the SBP and SFSBP groups was very similar to that of the sham group; slight subendocardial damage was observed, and a reduction of inflammatory cells was observed, which indicated that the SBP and SFSBP groups had experienced decreased myocardial damage. The fibrotic and necrotic effects were slightly reversed in the ginsenoside Rb1, ginsenoside Re, and cinnamic acid groups, which demonstrated that these 3 drugs showed therapeutic effects on MI, but they were not as effective as were the SBP and the SFSBP. Meanwhile, fibrotic and necrotic tissues were clearly observed in the borneol, bufalin, and muscone groups. All of the results demonstrated that the therapeutic performance was much lower in regulating myocardial damage when using the single components individually.

### 3.2. Method Validation for Stability and Repeatability of Sequence Analysis Using LC-Q-TOF/MS

The stability of sequence analysis using the LC-Q-TOF/MS system was validated by the variations in peak area and retention time of six extracted ions (*m/z* 269.05 (homocysteine, positive mode), *m/z* 118.07 (L-valine, positive mode), *m/z* 282.27 (oleamide, positive mode), *m/z* 608.3631 (PGPC, negative mode), *m/z* 188.0768 (3-indolepropionic acid, negative mode), and *m/z* 165.046 (3-methylxanthine, negative mode)). The relative standard deviations (RSDs) of the peak areas of the six extracted ions were all less than 13% in both the negative and the positive modes, while the relative standard deviations (RSDs) of the retention times of the six extracted ions were all less than 2% [14].

The repeatability of the LC-Q-TOF/MS system was validated by a reduplicated analysis of six parallel samples. The relative standard deviations (RSDs) of the peak areas of the six extracted ions were less than 15% in both the positive and the negative modes, while the relative standard deviations (RSDs) of the retentions times of the six extracted ions were all less than 1% [14].

All of the results demonstrated that the stability and the repeatability of the proposed method were satisfactory for the metabolomic study.

### 3.3. Metabolomics Study

#### 3.3.1. Biomarkers Contributed by MI

In our previous study, 8,302 ion signals in the positive mode and 7,289 ion signals in the negative mode were detected in both the MI model and the sham groups. Multivariate statistical analyses of a partial least squares discriminate analysis (PLS-DA) were performed by SIMCA-P software. Variable biomarkers were selected between the MI and the sham groups based on their variable importance in the projection threshold (VIP > 1). Eventually, 27 biomarkers (see Supplementary Table  1 available online at http://dx.doi.org/10.1155/2013/823121) were identified (14 in the positive and 13 in the negative) by searching through the Biofluid Metabolites Database (http://metlin.scripps.edu/) and the Human Metabolome Database (http://www.hmdb.ca/) for information on the MS and the MS/MS results in our previous study [14]. These biomarkers primarily involved 4 pathological pathways. Five biomarkers, including homocysteine [28–31], PGPC [32–34], 5-methylcytosine [35], hippuric acid [36], and allantoin [37], were related to oxidative injury from myocardial dysfunction. Five biomarkers, including AIR, hypoxanthine [38, 39], allantoin, lactic acid [40–42], and 3-methylxanthine, participated in energy metabolism dysfunctions induced by MI. Three biomarkers, including PGE2, leukotriene A4 methyl ester, and 12(S)-HETE, which are found in arachidonic acid metabolism, were involved in the inflammation that relates to MI. Five biomarkers, including L-proline, L-isoleucine, homocysteine, pyroglutamic acid, and L-valine, are amino acids which indicate that the formation of a MI has broken the balance of the amino acid metabolism. 

In this study, the different therapeutic effects of the 9 treatment groups have been evaluated through monitoring the changes in the 27 identified biomarkers.

#### 3.3.2. Different Therapeutic Effects of SFSBP and Its 7 Constituent Groups on MI

**Figure 3:**
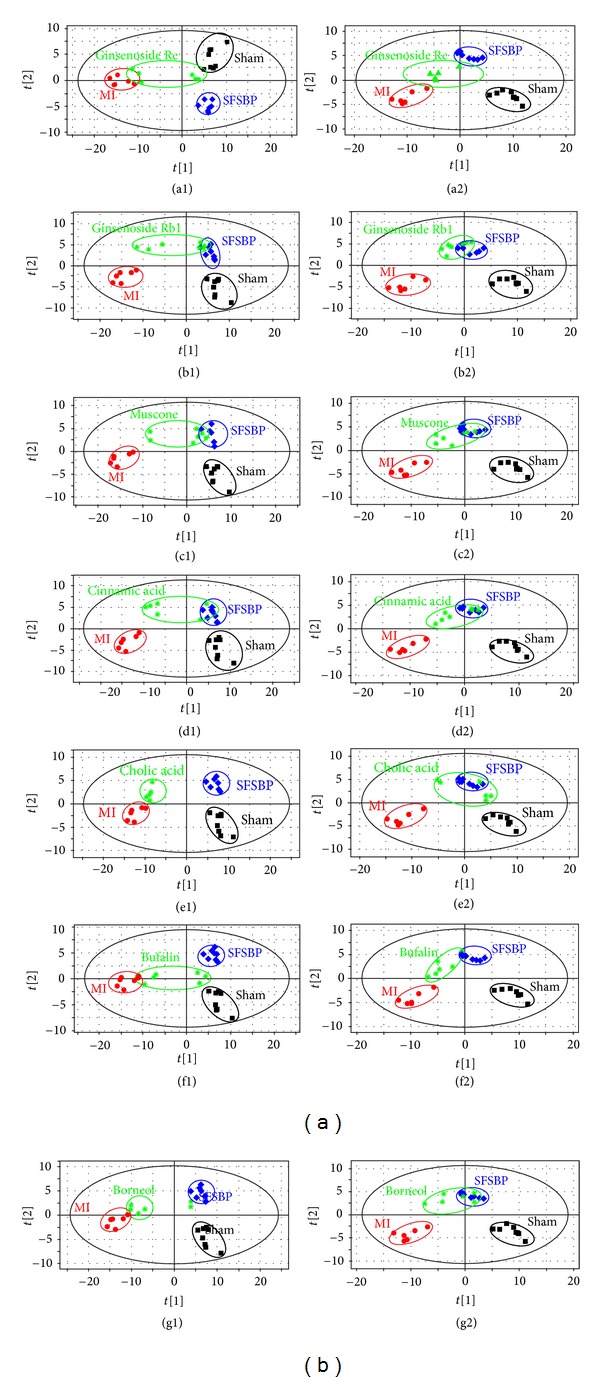
PLS-DA score plots of rat serum on day 15 in both positive and negative modes. (Parameters of each score plot were shown in Supplementry Material.)

The PLS-DA score plots (Figure 3) that were built to holistically evaluate the regulatory effects of SFSBP and its constituent treatment groups on MI in both positive and negative modes showed clear results. Although some of the monotherapy groups (ginsenoside Rb1, ginsenoside Re, bufalin, cinnamic acid, muscone, borneol and cholic acid treated groups) overlapped slightly with the SFSBP-group, none of the monotherapy groups were located closer to the sham group than the SFSBP-treated group was in either mode.

**Figure 4:**
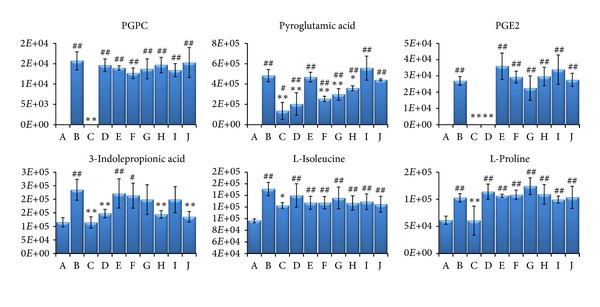
The six biomarkers that were regulated differently in SFSBP and its constituent groups. (A) sham group, (B) MI group, (C) SFSBP group, (D) ginsenoside Rb1, (E) ginsenoside Re, (F) bufalin, (G) cinnamic acid, (H) muscone, (I) borneol, and (J) cholic acid. *P < 0.05 and **P < 0.01 when compared with MI group; ^#^ *P *< 0.05, ^##^ *P *< 0.01 when compared with sham group. The number on the *y-axis *represents the peak areas of these biomarkers.

**Figure 5:**
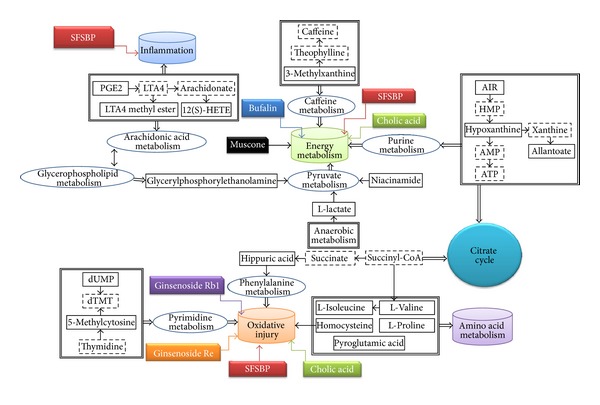
The distribution of regulation effects using different treatments. The solid-line squares represent the identified biomarkers; the cylinders represent pathogenesis induced by MI; the dashed squares represent different treatments.

The mean levels of the 27 identified biomarkers were also analyzed to evaluate the therapeutic effects of SFSBP and its 7 monocomponents. Nineteen of the biomarkers were significantly reduced to the level of the sham group by SFSBP, while the number of biomarkers reduced by ginsenoside Rb1, ginsenoside Re, bufalin, cinnamic acid, muscone, borneol, and cholic acid was 8, 6, 6, 8, 11, 5, and 6, respectively. As shown in Figure 5, the therapeutic effects of ginsenoside Rb1, ginsenoside Re, and cholic acid were primarily focused on regulating the biomarkers that relate to the oxidative injury (hippuric acid, homocysteine, and 5-methylcytosine) from MI, while bufalin and cinnamic acid were primarily focused on inhibiting the dysfunctions in energy metabolism (lactic acid) that are induced by MI. Furthermore, muscone achieved a significant therapeutic effect on the regulation of the oxidative injury (hippuric acid and 5-methylcytosine) and the dysfunctions in energy metabolism (lactic acid) related to MI, while borneol achieved scarcely any therapeutic effects on MI. Additionally, as shown in Figure 4, the mean levels of 2 biomarkers (pyroglutamic acid and 3-indolepropionic acid) in the SFSBP-treated group were reduced closer to the level of the sham group than they were in the monotherapy groups. Meanwhile, 4 biomarkers, including PGPC, PGE2, L-isoleucine, and L-proline were only significantly reduced to the level of the sham group by SFSBP. PGPC and PGE2 were involved in the oxidative injury and the inflammation from MI, respectively, indicating that the therapeutic effects of SFSBP came primarily from regulating the oxidative injury and the inflammation induced by MI.

All of the results indicated that SFSBP showed a stronger and more stable therapeutic effect for treating myocardial injury than did the monotherapies. Although each single therapy treated group was dosed at a higher dose concentration than was the SFSBP group, the SFSBP group showed the highest degree of recovery among the 8 treated groups. These results were consistent with pharmacology results that suggested that SFSBP could amplify the therapeutic effects of each of its constituents and exert synergistic therapeutic efficacies.

#### 3.3.3. Evaluation of Therapeutic Effectiveness of SFSBP on MI by Comparison with the SBP

**Figure 6:**
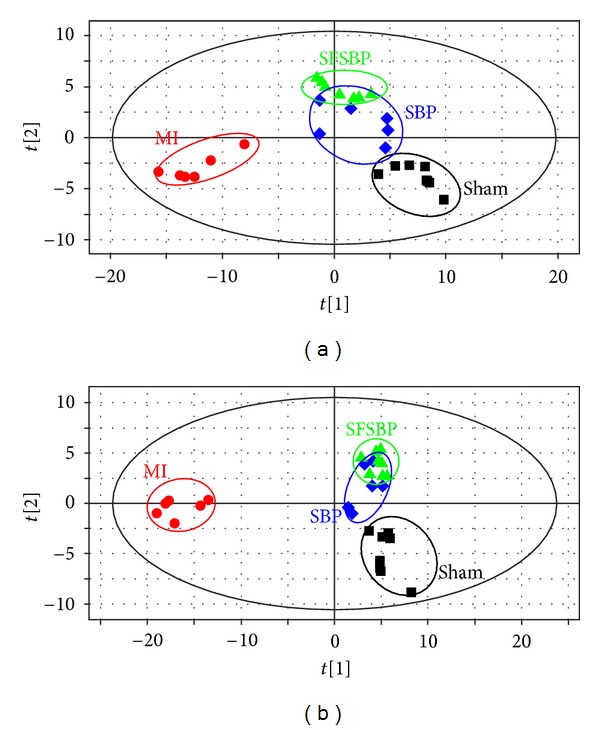
PLS-DA score plots of rat serum on day 15 in positive and negative modes. (a) PLS-DA score plot of rat serum of the SFSBP group in positive mode (*R* ^2^ *X*_(cum)_ = 0.596, *R* ^2^ *Y*_(cum)_ = 0.977, *Q* ^2^ *Y*_(cum)_ = 0.573); (b) PLS-DA score plot of rat serum of the SFSBP group in negative mode (*R* ^2^ *X*_(cum)_ = 0.516, *R* ^2^ *Y*_(cum)_ = 0.975, *Q* ^2^ *Y*_(cum)_ = 0.583).

Another PLS-DA model was built to evaluate the therapeutic effectiveness of the SFSBP and the SBP on MI. As shown in Figure 6, the SBP and the SFSBP groups were far from the MI group on the PLS-DA score plots. The SBP-treated group was closer to the sham group than it was to the SFSBP-treated group. Overlap between the two groups was also observed, revealing that the SBP achieved more effective therapeutic performance during the recovery from the MI surgery than did the SFSBP. 

**Figure 7:**
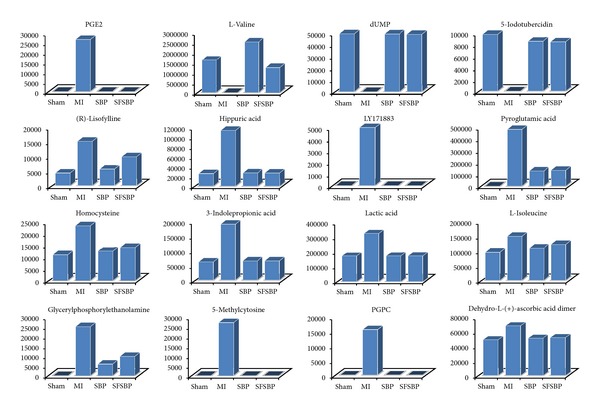
The sixteen biomarkers that were significantly reduced by both SBP and SFSBP. The number on the *y-axis* represents the peak areas of these biomarkers.

**Figure 8:**
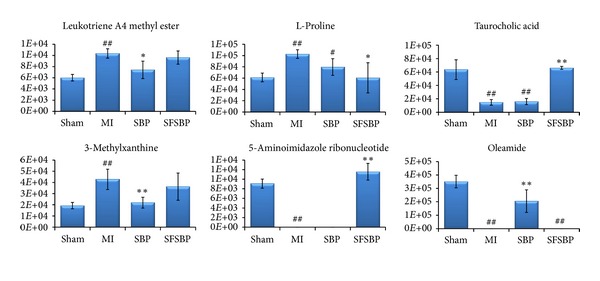
The six biomarkers that were differently regulated between the SBP- and SFSBP-treated groups. *P < 0.05 and **P < 0.01 when compared with the MI group; ^#^ *P *< 0.05 and ^##^P < 0.01 when compared with the sham group. The number on the *y-axis *represents the peak areas of these biomarkers.

The mean levels of the 27 identified biomarkers were also examined to evaluate the therapeutic effectiveness of the SFSBP and the SBP on MI. Nineteen biomarkers were significantly (*P *> 0.05, compared to sham group) reduced to the level of the sham group by either the SBP or the SFSBP. Among these biomarkers, 16 biomarkers (Figure 7) were reduced to the level of the sham group in both groups. Leukotriene A4 methyl ester, oleamide, and 3-methylxanthine (Figure 8) were reduced to the level of the sham group by the SBP (*P *> 0.05, SBP-treated group versus sham group), while L-proline, 5-aminoimidazole ribonucleotide, and taurocholic acid (Figure 8) were regulated to the level of the sham group in the SFSBP-treated group (*P *> 0.05, SFSBP-treated group versus sham group), and the remaining five biomarkers, hypoxanthine, niacinamide, 12(S)-HETE, 2-furoic acid, and allantoin, were not able to be restored by either the SBP or the SFSBP treatments. 

Among those biomarkers that were significantly reduced by the SBP and the SFSBP, lactic acid is involved in energy metabolism, hippuric acid, homocysteine, 5-methylcytosine, and PGPC are involved in oxidative injury, and PGE2 is involved in inflammation, which indicates that the SBP and the SFSBP might offer therapeutic effects on the dysfunctions in energy metabolism, oxidative injury, and inflammation induced by MI. Additionally, leukotriene A4 methyl ester (involved in inflammation) and 3-methylxanthine (involved in energy metabolism) can be reduced to the level of the sham group by the SBP but not by the SFSBP (Figure 8), indicating that the therapeutic effectiveness of the SBP may be superior to that of the SFSBP in inhibiting the inflammation and the dysfunctions in energy metabolism. 

The results demonstrated that the SBP and the SFSBP did have significant therapeutic efficacy in the MI rats and that their therapeutic mechanism was focused on inhibiting the dysfunctions in energy metabolism, oxidative injury, and inflammation from MI. Considering the fact that the SFSBP has the composition of the SBP, we can conclude that the SBP might also have synergistic effects in MI rats. However, the regulatory efficacy of the SBP on MI rats was superior to that of the SFSBP, according to the PLS-DA score plots. One of the reasons for this might be that there are other bioactive compounds in the SBP exerting therapeutic effects on MI, so that the composition of SFSBP needs to be further optimized. In consideration of the fact that the concentrations of 7 bioactive compounds in the SFSBP were much higher than those in the SBP, the therapeutic efficacy of the SFSBP was not as strong as that of the SBP, indicating the rationality of the TCM formula and the likelihood that it cannot simply be substituted with a combination of several bioactive compounds from its formula.

## 4. Conclusions

In this study, a metabolomic strategy has been performed to investigate the therapeutic and the synergistic effects of a TCM formula. This study revealed that the therapeutic effects of multicomponent medicines were greater than the effects of single components and that they exert a synergistic effect, in accordance with our previous study [14], which also indicated that TCM did have advantages in regulating the dysfunctions induced by complicated diseases. Finally, we believe that such a metabolomic-based approach is an efficacious strategy for the study of TCM.

## Supplementary Material

Table1: Twenty seven biomarkers have been identified in both positive and negative modes. These biomarkers were mainly involved in 4 pathological pathways (oxidative injury, dysfunction of energy metabolism, inflammation and dysfunction of amino acid metabolism).
