# Supplementary material for: Metabolomic Strategy for Studying the Intervention and the Synergistic Effects of the Shexiang Baoxin Pill for Treating Myocardial Infarction in Rats
Source: Evid Based Complement Alternat Med. 2013 Feb 28;2013:823121. doi: 10.1155/2013/823121 (PMC3603319; doi:10.1155/2013/823121)
Supplement: Supplementary file 1 — Table1: Twenty seven biomarkers have been identified in both positive and negative modes. These biomarkers were mainly involved in 4 pathological pathways (oxidative injury, dysfunction of energy metabolism, inflammation and dysfunction of amino acid metabolism). [file 823121.f1.doc]

**Table 1 Twenty seven identified biomarkers and their contribution to MI.**

| No. | Mass  (m/z) | Formula | Trend | Compound | Contribution to MI |
| --- | --- | --- | --- | --- | --- |
| 1 | 269.05 | C_8_H_16_N_2_O_4_S_2_ | ↑ | homocystine | Oxidative injury |
| 2 | 608.36 | C_29_H_56_NO_10_P | ↑ | PGPC | Oxidative injury |
| 3 | 126.05 | C_5_H_7_N_3_O | ↑ | 5-methylcytosine | Oxidative injury |
| 4 | 180.06 | C_9_H_9_NO_3_ | ↑ | hippuric acid | Oxidative injury |
| 5 | 157.04 | C_4_H_6_N_4_O_3_ | ↓ | allantoin | Oxidative injury and dysfunction of energy metabolism |
| 6 | 294.05 | C_8_H_14_N_3_O_7_P | ↓ | 5-aminoimidazol ribonucleotide | Dysfunction of energy metabolism |
| 7 | 137.04 | C_5_H_4_N_4_O | ↓ | hypoxanthine | Dysfunction of energy metabolism |
| 8 | 89.03 | C_3_H_6_O_3_ | ↑ | lactic acid | Dysfunction of energy metabolism |
| 9 | 167.04 | C_6_H_6_N_4_O_2_ | ↑ | 3-methylxanthine | Dysfunction of energy metabolism |
| 10 | 351.22 | C_20_H_32_O_5_ | ↑ | PGE2 | inflammation |
| 11 | 319.24 | C_20_H_32_O_3_ | ↑ | 12(S)-HETE | inflammation |
| 12 | 333.23 | C_21_H_32_O_3_ | ↓ | leukotriene A4 methyl ester | inflammation |
| 13 | 116.06 | C_5_H_9_NO_2_ | ↑ | L-proline | Dysfunction of amino acid metabolism |
| 14 | 130.04 | C_5_H_7_NO_3_ | ↑ | pyroglutamic acid | Dysfunction of amino acid metabolism |
| 15 | 118.07 | C_5_H_11_NO_2_ | ↓ | L-valine | Dysfunction of amino acid metabolism |
| 16 | 130.09 | C_6_H_13_NO_2_ | ↓ | L-isoleucine | Dysfunction of amino acid metabolism |
| 17 | 216.05 | C_5_H_14_NO_6_P | ↑ | glycerylphosphorylethanolamine | Not known |
| 18 | 123.04 | C_6_H_6_N_2_O | ↓ | niacinamide | Not known |
| 19 | 319.16 | C_16_H_22_N_4_O_3_ | ↑ | LY171883 | Not known |
| 20 | 307.04 | C_9_H_13_N_2_O_8_P | ↓ | deoxyuridine monophosphate (dUMP) | Not known |
| 21 | 514.28 | C_26_H_45_NO_7_S | ↓ | taurocholic acid | Not known |
| 22 | 281.15 | C_13_H_20_N_4_O_3_ | ↑ | (R)-lisofylline | Not known |
| 23 | 113.01 | C_5_H_4_O_3_ | ↓ | 2-furoic acid | Not known |
| 24 | 282.27 | C_18_H_35_NO | ↓ | oleamide | Not known |
| 25 | 390.99 | C_11_H_13_IN_4_O_4_ | ↓ | 5-iodotubercidin | Not known |
| 26 | 347.02 | C_12_H_12_O_12_ | ↑ | dehydro-L-(+)-ascorbicacid dimer | Not known |
| 27 | 188.07 | C_11_H_11_NO_2_ | ↑ | 3-indolepropionic acid | Not known |

**PLS-DA score plots parameters of Fig. 3**

(A1) PLS-DA score plot of rat serum of ginsenoside Rb1 group (R^2^X_(cum)_ = 0.519, R^2^Y_(cum)_ = 0.976, Q^2^Y_(cum)_ = 0.609), positive; (A2) PLS-DA score plot of rat serum of ginsenoside Rb1 group (R^2^X_(cum)_ = 0.634, R^2^Y_(cum)_ = 0.985, Q^2^Y_(cum)_ = 0.75), negative; (B1) PLS-DA score plot of rat serum of ginsenoside Re group (R^2^X_(cum_) = 0.519, R^2^Y_(cum)_ = 0.988, Q^2^Y_(cum)_ = 0.828), positive; (B2) PLS-DA score plot of rat serum of ginsenoside Re group (R^2^X_(cum)_ = 0.617, R^2^Y_(cum)_ = 0.972, Q^2^Y_(cum)_ = 0.79), negative; (C1) PLS-DA score plot of rat serum of bufalin group (R^2^X_(cum)_ = 0.499, R^2^Y_(cum)_ = 0.988, Q^2^Y_(cum)_ = 0.752), positive; (C2) PLS-DA score plot of rat serum of bufalin group (R^2^X_(cum)_ = 0.565, R^2^Y_(cum)_ = 0.941, Q^2^Y_(cum)_ =0.682), negative; (D1) PLS-DA score plot of rat serum of cinamic acid group (R^2^X_(cum)_ = 0.497, R^2^Y_(cum)_ = 0.984, Q^2^Y_(cum)_ = 0.716), positive; (D2) PLS-DA score plot of rat serum of cinamic acid group (R^2^X_(cum)_ = 0.617, R^2^Y_(cum)_ = 0.963, Q^2^Y_(cum)_ = 0.671), negative; (E1) PLS-DA score plot of rat serum of muscone group (R^2^X_(cum)_ = 0.498, R^2^Y_(cum)_ = 0.989, Q^2^Y_(cum)_ = 0.766), positive; (E2) PLS-DA score plot of rat serum of muscone group (R^2^X_(cum)_ = 0.608, R^2^Y_(cum)_ = 0.969, Q^2^Y_(cum)_ = 0.764) , negative; (F1) PLS-DA score plot of rat serum of broneol group (R^2^X_(cum)_ = 0.51, R^2^Y_(cum)_ = 0.984, Q^2^Y_(cum)_ = 0.738), positive; (F2) PLS-DA score plot of rat serum of broneol group (R^2^X_(cum)_ = 0.606, R^2^Y_(cum)_ = 0.979, Q^2^Y_(cum)_ = 0.699), negative; (G1) PLS-DA score plot of rat serum of cholic acid group (R^2^X_(cum)_ = 0.521, R^2^Y_(cum)_ = 0.981, Q^2^Y_(cum)_ = 0.728), positive; (G2) PLS-DA score plot of rat serum of cholic acid group (R^2^X_(cum)_ = 0.62, R^2^Y_(cum)_ = 0.985, Q^2^Y_(cum)_ = 0.81), negative.
